# 

**Published:** 1878-01

**Authors:** 


PATRONS OF THIS JOURNAL will please remember that all business connected with subscriptions and advertisements should be arranged with the Publisher, H. H. DICKSON, and all communications furnishing reading matter for the JOURNAL should be addressed to the Editor.

